# Pharmacokinetics and metabolism of ketoconazole after single ocular instillation in Sprague-Dawley rats

**DOI:** 10.5599/admet.2387

**Published:** 2024-11-09

**Authors:** Jiang Pu, Jinsong He, Ru Xue, Ruiqi Gao, Yaoming Yu, Wanyong Feng

**Affiliations:** Bioduro-Sundia, Shanghai, China

**Keywords:** Plasma, cornea, retina, quantitation, metabolite identification, mass spectrometry

## Abstract

**Background and purpose:**

Ketoconazole is limited to its conditioned oral use due to hepatic toxicity. Its ocular eye drop administration may be an option for mycotic keratitis treatment. Therefore, it is necessary to explore its pharmacokinetic and metabolic profile via topical ocular administration.

**Experimental approach:**

Nine rats were dosed at 300 μg/rat via topical ocular administration, and sacrificed at 5, 30, and 120 min with 3 rats/timepoint. Plasma, cornea, retina, and vitreous humour samples were collected, processed, and analysed.

**Key results:**

Ketoconazole was quantified with a mean peak plasma concentration of 445 ng/mL at 5 min post-dose. In the rat ocular tissue, the mean ketoconazole concentration at 5 min post-dose was 423 μg/g in the cornea, 4.96 μg/g in the retina, and 1.19 μg/g in the vitreous humour, respectively. The mean ketoconazole concentration in each matrix decreased from 5 to 120 min. The mean ketoconazole concentration at 120 min was 38.4 ng/mL in plasma, and 8.36, 0.0944, and 0.116 μg/g in the cornea, retina, and vitreous humour, respectively. Pooled plasma, cornea, retina, and vitreous humour homogenates were used for metabolite identification. Nine metabolites were identified in rat plasma, and O-dealkylated metabolite (M3) and dehydrogenated metabolite (M11) were the top two, accounting for 5.0 and 5.8 % of the relative mass abundance. The metabolic pathways were O-dealkylation, mono-oxygenation, and dehydrogenation. Eleven metabolites were identified in the rat cornea, and two metabolites were identified in the rat retina and vitreous humour, respectively. The O-dealkylated and hydrogenated metabolite (M2) was a dominant metabolite in the cornea, retina, and vitreous humour, while M3 and M11 were the dominant metabolites in plasma.

**Conclusion:**

Ketoconazole was a dominant component (≥ 98.5 %) in the cornea, retina, and vitreous humour, having higher concentrations in cornea than in plasma. M2 was identified as a dominant metabolite (1.1-1.2 %) in the cornea, retina, while M3 (5.0 %) and M11 (5.8 %) were identified as dominant metabolites in the plasma.

## Introduction

Mycotic keratitis is a common disease in ophthalmology. Its symptoms are not typical in the early stages. *Fusarium* and *Curvularia* are the major pathogens in river districts, and once infected, they could severely affect vision. Ketoconazole is a drug used in the management and treatment of fungal infections. As a dioxolane imidazole [1,2], ketoconazole inhibits sterol biosynthesis in the fungal membrane [3]. This inhibition could deprive the fungal cell of its protective sterol membrane, making ketoconazole widely accepted and used as a broad-spectrum drug to treat mycotic keratitis.

Ketoconazole is a weak dibasic agent and requires acidity for dissolution and absorption. Its pharmacokinetics in humans has been widely studied and characterized. It reaches its maximum plasma concentration at 1-2 h and exhibits a biphasic elimination of 2 h during the first 10 h and 8 h thereafter [4,5]. CYP3A4 is the major enzyme involved in the metabolism of ketoconazole. The major identified metabolic pathways are oxidation and degradation of the imidazole and piperazine rings by hepatic microsomal enzymes. In addition, oxidative *O*-dealkylation and aromatic hydroxylation do occur. Many drug-drug interaction studies of ketoconazole *in vitro* and *in vivo* human systems have been reported, and such information is well documented [4,5]. Ketoconazole has also been recognized as a specific CYP3A4/5 inhibitor and a strong clinical CYP3A4 inhibitor, as well as an inhibitor of P-gp, BCRP, OATPs, OAT3, OCT2, and MATEs transporters, clinical P-gp inhibitor for drug-drug interaction studies recommended by regulatory agencies [6]. Orally administered ketoconazole is limited in clinical use due to metabolism-related hepatotoxicity [7-10]. *N*-deacetyl ketoconazole (DAK), as a primary metabolite of ketoconazole, is believed to undergo metabolism through flavin-containing monooxygenase (FMO) and may be transformed into a potentially toxic dialdehyde [11-14]. Rodriguez *et al.* [11] studied the toxicity of DAK and demonstrated that it was more metabolically cytotoxic than ketoconazole. Afterwards, Haegler *et al.* [12] studied the mitochondrial toxic effects of ketoconazole at concentrations easily reached in vivo. They found that cytotoxicity and adenosine triphosphate (ATP) depletion were more pronounced in cells with mitochondrial damage. In July, 2013, the US FDA released a safety announcement regarding the use of oral ketoconazole tablets and their potential adverse drug reactions. It was advised that ketoconazole should not be used as a first-line treatment for fungal infections due to the risk of potentially serious drug-drug interactions [15-17]. The European Medicines Agency had also proposed limiting the use of oral ketoconazole in fungal infections due to the same risk [18,19].

Unlike oral administration, local drug administration, such as intramuscular, intraperitoneal, subcutaneous, transdermal, and topical ocular administration, can be an alternative approach to avoid first-pass intestinal and hepatic metabolism and associated drug-drug interactions and achieve higher exposure levels in target organs due to a lower metabolic loss of the drug. Ocular drug delivery has been an active area in the use of old drugs and new ophthalmic drug discovery and development [20-23]. Fundamental drug-metabolizing enzymes, uptake, and efflux transport information in different locations of the eyes in animals and humans (cornea, lens, iris, retina, vitreous humour, *etc*.) are very limited. The eye is a complex organ with protective anatomy and physiology. Understanding pharmacokinetics and drug metabolism in ocular tissues is a major challenge because of the complex anatomy and dynamic physiological barrier of the eye. Ocular drug metabolism in the eye is crucial for ocular therapeutics to facilitate the risk assessment and the development of potential drug candidates in the clinical stage. In the discovery stage, rabbits and other less common species, such as dogs, monkeys, and rats, are also used to study ocular pharmacokinetics and drug metabolism [24-28]. As shown in a recent mini-review, twenty-six enzymes, including eleven human CYP enzymes (CYP1A2, CYP1B1, CYP2A6, CYP2B6, CYP2C8, CYP2C9, CYP2C19, CYP2E1, CYP2D6, CYP3A4, CYP3A5) and other phase I/II enzymes in cornea, lens, iris, ciliary body, retina, choroid, retinal pigment epithelium, optic nerve and sclera, and 38 uptake and efflux transporters including MATE1-2, PEPT1-2, P-gp, BCRP, and MRP1-6 in cornea, lens, iris, ciliary body, retina and choroid, were reported [29].

Although the metabolism of ketoconazole after oral administration in humans is well studied, it is not well explored in the eye in any preclinical species or humans. In rats, ketoconazole has been well studied as a substrate *in vitro* system [30,31] and *in vivo* systems [30-32] or as a rat CYP3A inhibitor *in vitro* systems [33-35] and *in vivo* systems [36-57]. Cirello *et al.* [58] reported that in rat, rabbit, and human ocular and liver S9 fractions, eleven metabolites were identified, six metabolites were found in rat ocular, whereas eight were present in rabbit and human ocular S9 fractions. Three more metabolites were found in rat liver than in rat ocular S9 fractions. Metabolic pathways in rabbit and human ocular fractions suggested the formation of reactive intermediates in rabbit and human liver, and ocular S9 incubations. Bioactivation was found to occur via iminium ion in both human and rabbit ocular S9 fractions, and the reactive intermediates were identified using potassium cyanide trapping methodology. To our best knowledge, no *in vivo* ocular metabolism of ketoconazole in rats has been reported, although it has been extensively investigated following oral or intravenous administration in rats. To understand the metabolic disposition of ketoconazole in different eye tissues, we chose the rat to study the ocular metabolism of ketoconazole in plasma, cornea, retina, and vitreous humour after a single topical instillation administration.

## Experimental

### Chemicals and reagents

Ketoconazole (SLCC4160) was purchased from Sigma in USA; (2-Hydroxypropyl)-β-cyclodextrin (HPβCD, WXBC0083V) was also obtained from Sigma in USA; Distilled H_2_O (20190802 C) was purchased from Watsons in China.

### Formulation preparation

Ketoconazole was weighed and dissolved in a 4 mL glass vial, and then 0.083 mL of water was added and vortexed. The mixture was stirred at 40 °C. Subsequently, 0.040 mL of 1 M hydrochloric acid was added, and the mixture was stirred at 40 °C until it was completely dissolved. 0.250 mL of hydroxypropyl beta-cyclodextrin (HPβCD) was added and vortexed and stirred at 40 °C until clear. 0.005 mL of 1 M sodium hydroxide was added to adjust the pH to 6.5-7.0 while stirring magnetically at 40 °C, and 0.038 mL of water was added, vortexed, and stirred at 40 °C until clear. At last, a 30 mg/mL ketoconazole clear solution was prepared in 12.5 % HPβCD in water.

### Animal administration

All procedures performed on the animals were in accordance with regulations and established guidelines and were reviewed and approved by an Institutional Animal Care and Use Committee through an ethical review process. Nine male Sprague Dawley rats were 7 to 9 weeks old and purchased from Vital River (Zhejiang, China). Rats were allowed to acclimate approximately one week before treatment, with water and food available *ad libitum* throughout the acclimation and experimental periods.

Nine rats were randomly divided into three groups and their body weights were recorded from 254 to 281 g. A 5 μL ketoconazole solution (30 mg/mL in 12.5 % HPβCD ) was instilled in both eyes, delivering 150 μg per eye (total 300 μg per rat) over 30 seconds. Rats were sacrificed at 5, 30 and 120 min post-dose (*n* = 3 per time point). Cornea, retina, and vitreous humour samples were collected, washed in saline three times and weighed, while blood samples were taken from the heart puncture. Tissue samples were initially stored on dry ice, while the blood samples were centrifuged at 4 °C and 6000 rpm for 10 min. Subsequently, the plasma samples were prepared and also stored on dry ice. Ultimately, all samples were cryopreserved in a -80 °C freezer after harvest.

### Tissues homogenization and treatment

All tissue samples were removed from the -80 °C freezer, thawed on ice, and 0.200 mL of 75 % acetonitrile was added to each tissue. Each tissue was then homogenized using an Ultrasonic Cell Crusher for 5 seconds. The cornea and retina homogenates were sonicated for 10 min, while the vitreous was sonicated for 30 min at 0 °C prior to bioanalysis. The ocular tissue homogenates were stored in a -80 °C freezer before bioanalysis.

All samples underwent protein precipitation for pharmacokinetic analysis. Plasma and three types of tissue homogenate samples (50 μL each) were treated with 5 μL of methanol and then quenched with a 200 μL methanol and acetonitrile mixture (1:1, v/v) containing 50 ng/mL terfenadine as an internal standard (ISTD). After vortexing for 1 min and centrifuging at 4000 rpm for 15 min, the supernatant was transferred and diluted 10-fold with a methanol/water solution (1:1, v/v, containing 0.1 % formic acid) for LC-MS/MS analysis. The working solution was prepared with a concentration range of 50.0 to 100,000 ng/mL. The ketoconazole standard curve for corneal homogenates ranged from 5.00 to 10,000 ng/mL, whereas for retina and vitreous humour homogenates, it ranged from 1.00 to 2,000 ng/mL.

Rat plasma (400 RL, 44.4 LL/rat at each time point) was pooled from 3-time points utilizing the equal volume method and subsequently precipitated with acetonitrile containing 0.1 % formic acid at a ratio of 1:3. Homogenate samples of rat cornea, retina, and vitreous humour (600 L, 66.7 LL /rat) were pooled from three time points individually utilizing the equal volume and transferred into a 15 mL centrifuge tube and subjected to precipitation with acetonitrile containing 0.1 % formic acid at the same ratio of 1:3. Following centrifugation, the supernatant was evaporated under a stream of nitrogen gas. The residues from plasma and rat retina were reconstituted with 150 L of 10 % acetonitrile in H_2_O (with 0.1 % formic acid), while the residues from the rat cornea and vitreous humour were reconstituted with 100 L of 10 % acetonitrile in H_2_O (0.1 % formic acid). Four types of blank samples were processed in a similar manner. Ultimately, 10,L samples of each tissue were injected for analysis using the Ultimate 3000-Q-Exactive Plus system.

### Liquid chromatography-mass spectrometry analysis

The quantitative sample analysis was performed on a Shimadzu LC-20AD (Shimadzu, Japan) - API 4000 mass spectrometer (SCIEX, Canada). The chromatographic separation of analytes from the matrix was achieved on a Kinetex 2.6 μm C18 100A column (3 30 mm) at room temperature with a flow rate of 0.7 mL/min. For plasma analysis, mobile phase A consisted of 5 mM ammonium acetate in distilled water (with 0.05 % formic acid), and mobile phase B consisted of acetonitrile (with 0.1 % formic acid). A step time gradient started by maintaining 15 % mobile phase B for 0.4 min, transitioning from 15 to 95 % mobile phase B over 1.8 min, holding at 95 % mobile phase B for 0.3 min, then returning to 15 % B in 0.01 min and re-equilibrating for 0.49 min. For the cornea, retina, and vitreous humour, a step time gradient started by maintaining 15 % mobile phase B for 0.4 min, transitioning from 15 to 95 % mobile phase B over 0.8 min, maintaining 95 % mobile phase B for 0.6 min, then returning to 15 % B in 0.01 min and re-equilibrating for 0.69 min. Ketoconazole was detected and quantified by performing positive-ion electrospray tandem mass spectrometry at 4500 V and 550 °C, with multiple reaction monitoring modes of ion transition *m*/*z* 531.2 to 489.2 for ketoconazole and *m*/*z* 472.4 to 436.4 for terfenadine (ISTD).

The metabolite identification and analysis were conducted on an Ultimate 3000 - Q-Exactive plus high-resolution mass spectrometer (Thermo Fisher Scientific, Germany), ketoconazole and its metabolites were injected and separated using an ACQUITY UPLC® HSS T3 column (1.8 μm, 2.1×100 mm) at room temperature with a flow rate of 0.3 mL/min. Mobile phase A consisted of water containing 0.1 % formic acid, while mobile phase B consisted of acetonitrile with 0.1 % formic acid. Chromatographic separation was achieved with a step time gradient by maintaining 5 % mobile phase B for 1.0 min, increasing from 5 to 25 % mobile phase B over 5.0 min, further increasing from 25 to 40 % mobile phase B over 8.0 min, then from 40 to 95 % mobile phase B over 3.0 min, maintaining 95 % mobile phase B for 2.0 min, and finally returning to 5 % B in 0.1 min and re-equilibrating for 2.9 min. Mass scanning was performed on the Q-Exactive Plus using positive-ion full MS/dd-MS2 mode with a range from *m*/*z* 80 to 900. The collision energy was set at 30, 40 and 50 eV. The mass resolution was set as 70,000 for full MS, and 17,500 for dd-MS2 scans, and the mass error limit was set as 5.0 ppm.

## Results and discussion

### Pharmacokinetics characteristics

**Figure 1.**
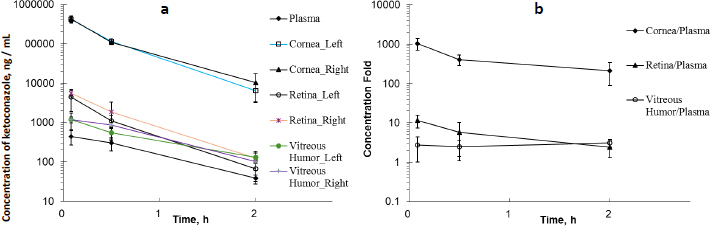
Ketoconazole concentration in ocular tissue and plasma (a) and ratios of ocular tissue to plasma concentration (b)

As shown in the left plot in Figure 1, ketoconazole concentrations were measured in plasma, cornea, retina, and vitreous humour at 5, 30 and 120 min after topical ocular administration in each eye. In rat plasma, the mean concentration was determined to be 0.445 μg/mL at 5 min and then decreased over time. In rat cornea, the mean concentration was found to be extremely high at 416 μg/g in the left eye and 430 μg/g in the right eye at 5 min post-dose. In rat retina samples, the mean concentration was 0.439 μg/g in the left eye and 0.552 μg/g in the right eye at 5 min post-dose. Similar to rat retina, the mean concentration in rat vitreous humour was 1.12 μg/g in the left eye and 1.18 μg/g in the right eye at 5 min post-dose. The mean ketoconazole concentration in each tissue decreased from 5 to 120 min. The mean concentration of left and right eye tissue at 30 min post-dose was 0.303 μg/mL in plasma, and 113, 1.47 and 0.713 μg/g in cornea, retina, and vitreous humour, respectively. At 120 min post-dose, it was 0.0384 μg/mL in plasma and 8.36, 0.0944 and 0.116 μg/g in cornea, retina, and vitreous humour, respectively.

Ketoconazole exhibited higher concentrations in the cornea, retina, and vitreous humour compared to plasma following topical ocular administration. As illustrated in Figure 1b, ketoconazole showed the highest tissue/plasma ratios in the cornea. Similarly, the retina and vitreous humour also demonstrated relatively elevated tissue/plasma ratios. This finding suggested that ketoconazole was readily accessible to the cornea following ocular administration. In an early study in rabbits [36], a low ocular tissue/serum ratio was observed following the oral administration of ketoconazole at 43.5 mg/kg. Ketoconazole serum concentrations ranged from 5.00 to 10.0 μg/mL, while corneal and vitreous humour concentrations were detectable and ranged from 0 to 0.0400 μg/mL within four hours post-dose, respectively. A different administration route may result in different ocular tissue/plasma or ocular tissue/serum ratios.

### Metabolites identification and profiling

**Figure 2.**
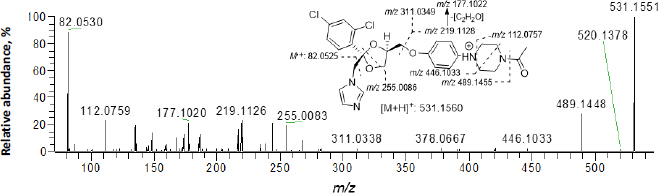
Collision-induced dissociation spectrum of ketoconazole (CE = 40 eV)

**Figure 3.**
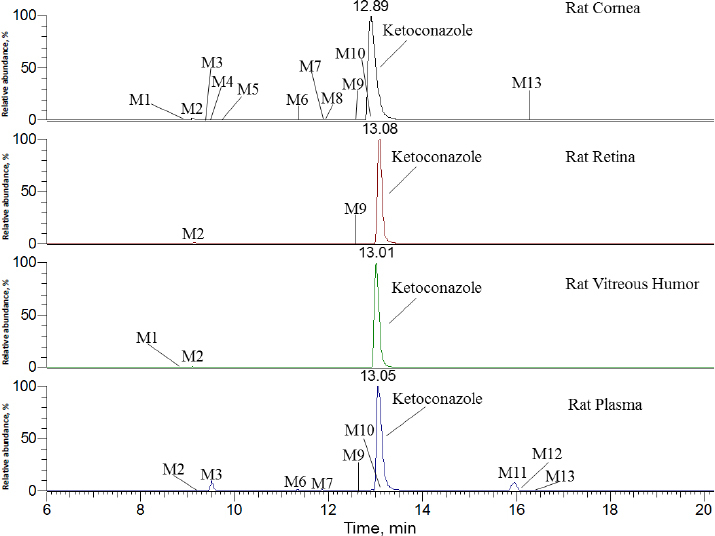
Selected ion chromatogram of ketoconazole and its metabolites

In Figure 2, ketoconazole showed a protonated molecular ion [M+H]^+^ at *m*/*z* 531.1551 (~1.9 ppm). The collision-induced dissociation (CID) product ion spectrum showed characteristic fragment ions *m*/*z* 82.0530, 112.0759, 177.1020, 219.1126, 255.0083, 311.0338, 446.1033 and 489.1448. The LC retention time was 13.06 min. As shown in Figure 3 and Table 1, a total of thirteen metabolites were eluted from 8.91 to 16.34 min and extracted within a mass error of 5 ppm, most metabolites were eluted earlier than ketoconazole and turned out to be much more polar compounds.

**Table 1.** Observed metabolites of ketoconazole in rat plasma and ocular tissues after eye drop administration

MET#	RT, min	*m*/*z* (+)		Mass error, ppm	Metabolite pathway	Relative peak area abundance, %				Ref.
		Theoretica	Detected			COR	RET	VH	PLA	
M1	8.91	547.1510	547.1500	-1.8	Mono-oxygenation	D	ND	D	ND	[55,56,58]
M2	9.16	329.0454	329.0446	-2.4	*O*-dealkylation and hydrogenation	1.1	1.2	0.6	0.1	[55,56]
M3	9.44	343.0247	343.0242	-1.5	*O*-dealkylation and mono-oxygenation	D	ND	ND	5.0	[56,58]
M4 (DAK)	9.56	489.1455	489.1449	-1.2	Hydrolysis	0.2	ND	ND	ND	[11,13,30,55,56,58]
M5	9.73	505.1404	505.1399	-1.0	Hydrolysis and mono-oxygenation	D	ND	ND	ND	[55,56,58]
M6	11.41	480.1451	480.1440	-2.3	Ring-opening *N*-dealkylation	0.1	ND	ND	0.6	[56]
M7	11.92	545.1353	545.1343	-1.8	Mono-oxygenation and dehydrogenation	D	ND	ND	0.6	[55,56,58]
M8	11.98	547.1510	547.1531	3.8	Mono-oxygenation	0.2	ND	ND	ND	[55,56,58]
M9	12.56	545.1353	545.1337	-2.9	Mono-oxygenation and dehydrogenation	D	D	ND	0.1	[55,56,58]
M10	13.05	547.1510	547.1496	-2.6	Mono-oxygenation	D	ND	ND	0.6	[55,56,58]
KCZ	13.06	531.1560	531.1550	-1.9	--	98.3	98.8	99.4	85.4	--
M11	15.83	529.1404	529.1390	-2.6	Dehydrogenation	ND	ND	ND	5.8	[55,58]
M12	15.88	545.1353	545.1343	-1.8	Mono-oxygenation and dehydrogenation	ND	ND	ND	1.6	[55,56,58]
M13	16.34	545.1353	545.1345	-1.5	Mono-oxygenation and dehydrogenation	0.1	ND	ND	0.2	[55,56,58]

Note: KCZ - Ketoconazole; MET# - metabolite identity number; COR - cornea; RET - retina; VH - vitreous humour; PLA - plasma; RT - retention time; D - detected in trace amount (the relative abundance was less than 0.1 %); ND - not detected; RT - retention time; DAK - deacetyl ketoconazole; The relative abundances of the parent and metabolites were calculated based on their selected ion chromatographic peak areas.

**Figure 4.**
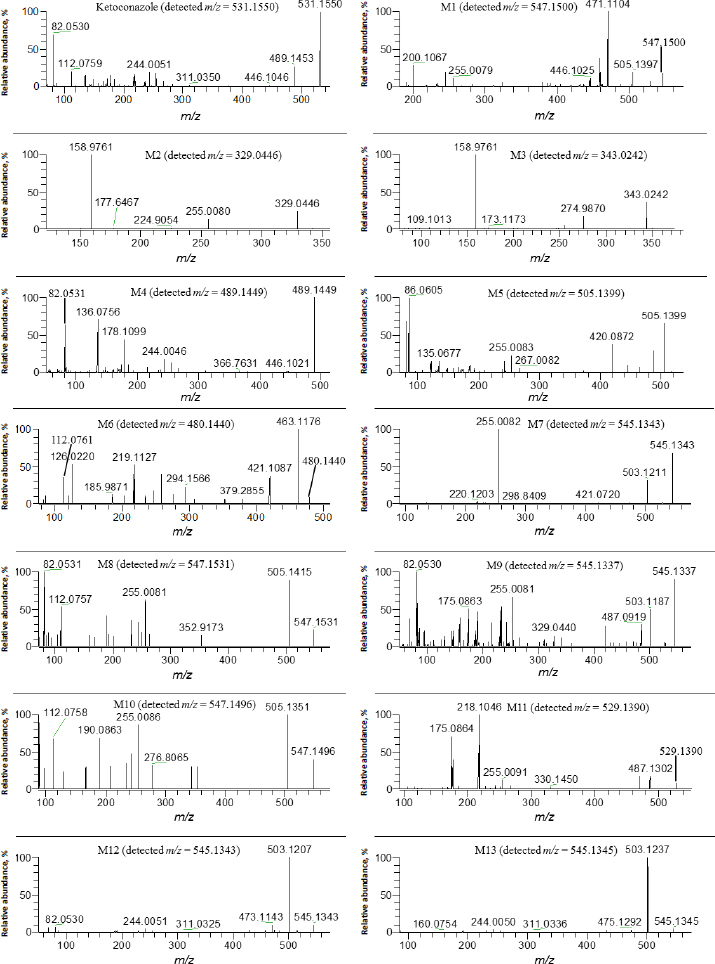
MS/MS spectra of metabolites of ketoconazole in rat cornea, retina, vitreous humour and plasma

Several metabolites were identified by the mass defect method based on the characteristic fragment ions compared with ketoconazole, as shown in Figure 4. M1 showed a protonated molecular ion at *m*/*z* 547.1500, its characteristic ions were *m*/*z* 255.0079, 471.1104 and 505.1397. M8 showed a protonated molecular ion at *m*/*z* 505.1496, its characteristic ion 505.1415. M10 showed a protonated molecular ion at *m*/*z* 547.1409, its characteristic ion was *m*/*z* 505.1351. The addition of 16 amu to parent fragment ions indicated M1, M8 and M10 were mono-oxygenation metabolites. M4 showed a protonated molecular ion at *m*/*z* 489.1449, its CID product ion spectrum displayed fragment ions at *m*/*z* 82.0531, 136.0756, 178.1099 and 446.1021, the loss of 42 amu from parent ion indicated it was a hydrolysis metabolite, also called as a deacetyl-ketoconazole (DAK). M5 showed a protonated molecular ion at *m*/*z* 505.1399, its CID product ion spectrum displayed fragment ions at *m*/*z* 255.0083, 267.0082 and 420.0872, the addition of 16 amu to M4 indicated it was a metabolite of hydrolysis and mono-oxygenation. M11 exhibited a protonated molecular ion at *m*/*z* 529.1390, its CID product ion spectrum displayed fragment ions at *m*/*z* 175.0864, 218.1046, 255.0091 and 487.1302, the loss of 2 amu from parent fragment ions indicated it was a dehydrogenation metabolite.

M7 showed a protonated molecular ion at *m*/*z* 545.1343, its CID product ion spectrum displayed fragment ions at *m*/*z* 255.0082, 421.0720 and 503.1211. M9 showed a protonated molecular ion at *m*/*z* 545.1337, its CID product ion spectrum displayed fragment ions at *m*/*z* 255.0081, 329.0440, 487.0919 and 503.1187 M12 showed a protonated molecular ion at *m*/*z* 545.1343, its CID product ion spectrum displayed fragment ions at *m*/*z* 244.0051, 311.0325 and 503.1207. M13 exhibited a protonated molecular ion at *m*/*z* 545.1345, its CID product ion spectrum displayed fragment ions at *m*/*z* 244.0050, 311.0336 and 503.1237. The addition of 14 amu to parent fragment ions indicated that M7, M9, M12, and M13 were metabolites of mono-oxygenation and dehydrogenation.

The other metabolites were elucidated according to their characteristic fragment ions and *m*/*z* accuracy. M2 showed a protonated molecular ion at *m*/*z* 329.0446, its CID product ions were *m*/*z* 158.9761 and 274.9870, the loss of 202 amu from parent ion indicated it was a metabolite of *O*-dealkylation and hydrogenation, this metabolite was also determined in rat, rabbit and human liver S9 fractions by Cirello *et al.* [55] according to the same fragment (*m*/*z* 255.0086). M3 showed a protonated molecular ion at *m*/*z* 343.0242, its CID product ion spectrum showed fragment ions at *m*/*z* 158.9763, 255.0086 and 274.9870, the loss of 188 amu to the parent ion, indicating that it was a metabolite of *O*-dealkylation and mono-oxygenation. M6 showed a protonated molecular ion at *m*/*z* 480.1440, its CID product ion spectrum displayed fragment ions at *m*/*z* 112.0761, 219.1127, 421.1087 and 463.1176, the loss of 51 amu from parent ion indicated it was a metabolite of ring-opening *N*-dealkylation.

As indicated in Figure 3 and Table 1, ketoconazole was slowly eliminated in pooled plasma, cornea, retina, and vitreous humour samples within 120 min after topical ocular administration, and it was still a dominant component. In rat plasma, nine metabolites of ketoconazole were identified, the top two metabolites were M3 and M11, accounting for 5.0 and 5.8 % of the total mass abundance, its biotransformation involved *O*-dealkylation, mono-oxygenation and dehydrogenation. In the rat cornea, eleven metabolites were identified. The primary metabolite was M2, which underwent biotransformation through *O*-dealkylation and hydrogenation. In the rat retina, two metabolites were identified, with M2 being the top one. In the vitreous humour, two metabolites were identified, with M2 remaining the primary one.

**Figure 5.**
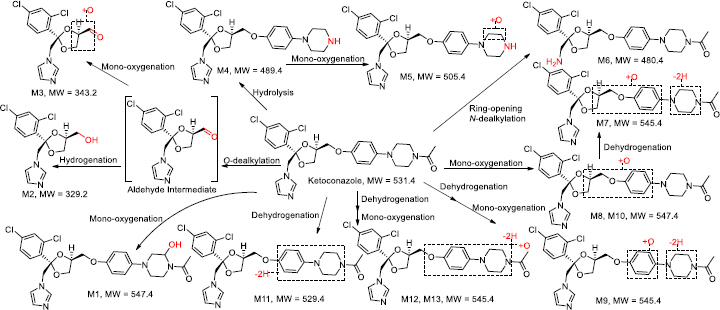
Proposed metabolic pathways of ketoconazole in rat plasma and ocular tissues

As summarized in Figure 5 and Table 1, observed metabolic pathways included *O*-dealkylation, hydrogenation, mono-oxygenation, hydrolysis, and dehydrogenation, consistent with previous studies [13,55]. Interestingly, an aldehyde metabolite labelled as an intermediate via *O*-dealkylation, which is responsible for the subsequent hydrogenation to M2 and mono-oxygenation to M3, was not detected in this study. In Cirello’s study [55], “M10” (*m*/*z* 329.0456) has a fragment (*m*/*z* 255.0081) and the comparison suggests that “M10” is equivalent to M2 (*m*/*z* 329.0454) with its fragment ions (*m*/*z* 158.9761, 255.0080) of this study. In addition, in Fitch’s study [56], “M16” (*m*/*z* 343.0256) has three fragment ions (*m*/*z* 159. 275, 255) and the comparison suggests that “M16” is equivalent to M3 (*m*/*z* 343.0247) with its fragment ions (*m*/*z* 158.9761, 274.9870, 255.0087) of this study. M3 in this study was also identified in Kim’s work [58] as “M14” (*m*/*z* 343.0247). In early reports [11-14], *N*-deacetyl ketoconazole was also identified as M4, and it possessed a bioactivation function that could potentially be transformed into a cytotoxic agent. However, it was not detected in rat plasma, suggesting that the hepatic toxicity potential is unlikely to occur in rats due to the low systemic concentration after ocular administration. Ketoconazole was a dominant component in the cornea, retina, and vitreous humour. This may suggest a better fit for topical usage in rats and, eventually, in human topical ocular use.

## Conclusion

Ketoconazole exhibited higher concentrations in the cornea, retina and vitreous humour than in plasma in rats following single topical ocular administration. The mean ketoconazole concentration in each tissue decreased from 5 to 120 min. The observed metabolic pathways were *O*-dealkylation, mono-oxygenation, and dehydrogenation. Nine metabolites were identified in rat plasma, and the *O*-dealkylated metabolite (M3) and dehydrogenated metabolite (M11) were top two abundant metabolites, accounting for 5.0 and 5.8 % of the total mass abundance, respectively. Eleven metabolites were identified in the rat cornea, and two metabolites were detected in the rat retina and vitreous humour, respectively. The *O*-dealkylated and hydrogenated metabolite (M2) was a dominant metabolite in the cornea, retina, and vitreous humour, while M3 and M11 were the dominant metabolites in plasma. No *N*-deacetyl ketoconazole (M4) was found in the plasma.
